# Rosaï-Dorfman-Destombes Disease: A Clinical Case Report

**DOI:** 10.7759/cureus.76934

**Published:** 2025-01-05

**Authors:** Nourelhouda Ouerradi, Chaimae N'joumi, Ayyad Ghannam, Aziza Elouali, Abdeladim Babakhouya, Maria Rkain

**Affiliations:** 1 Department of Pediatrics, University Health Centre (UHC) Mohammed VI, Oujda, MAR; 2 Faculty of Medicine and Pharmacy of Oujda, Mohammed I University, Oujda, MAR; 3 Department of Pediatric Gastroenterology, University Health Centre (UHC) Mohammed VI, Oujda, MAR

**Keywords:** cervical lymphadenopathy, management, post-viral, rosai-dorfman-destombes disease, sinus histiocytosis with massive lymphadenopathy

## Abstract

Rosaï-Dorfman disease (RDD), also known as sinus histiocytosis with massive lymphadenopathy (SHML), is a rare and benign histoproliferative disorder of unknown etiology. It commonly presents with bilateral, painless cervical lymphadenopathy accompanied by systemic features such as fever and leukocytosis. RDD manifests in two primary forms: a systemic form involving multiple organ systems and a cutaneous form confined to the skin. Histopathological examination typically reveals pericapsular fibrosis, sinusoidal dilation, and the presence of large histiocytes exhibiting emperipolesis (phagocytosed lymphocytes within histiocytes). Extranodal involvement is observed in approximately 43% of the cases, with a predilection for the head and neck regions. Although the precise etiology remains unclear, associations with viral infections such as Epstein-Barr virus (EBV) and human herpesvirus-6 (HHV-6) have been proposed. Management strategies are case-dependent, ranging from observation in cases of spontaneous remission (reported in 20-50% of patients) to more aggressive therapeutic interventions. Here, we describe a pediatric case of RDD managed in Morocco.

## Introduction

Rosaï-Dorfman-Destombes disease (RDD), also known as sinus histiocytosis with massive lymphadenopathy (SHML), is a benign systemic histoproliferative disorder of unknown etiology. Clinically, it presents with bilateral painless cervical lymphadenopathy, fever, and leukocytosis identified on blood examination. RDD is classified into two clinical forms: a systemic form and a cutaneous form. The systemic form, consistent with the traditional definition of the disease, involved multiple organ systems and was first described by Destombes in 1965. It was later recognized as a distinct pathological entity by Rosaï and Dorfman in 1969. While sharing the same histopathological characteristics, the cutaneous form differs in its epidemiological profile and is confined solely to the skin, with no systemic involvement [1].

Histopathological analysis of RDD typically reveals pericapsular fibrosis, sinusoidal dilation, and the presence of large histiocytes with emperipolesis (intact phagocytized lymphocytes within the cytoplasm). A dense inflammatory infiltrate of histiocytes, lymphocytes, and plasma cells is also characteristic [2].

RDD should be considered in the differential diagnosis of cervical lymphadenopathy, and heightened awareness among clinicians and pathologists is essential. Although the etiology remains idiopathic, RDD has been associated with infections, particularly viral, including Epstein-Barr virus (EBV), human herpesvirus 6 (HHV-6), parvovirus B19, and polyomavirus. These associations are supported by immunohistochemical, polymerase chain reaction (PCR), and in situ hybridization studies [3].

Extranodal involvement is observed in approximately 43% of the RDD cases, most commonly in the head and neck region, with manifestations morphologically similar to nodal RDD. Extranodal lesions may also involve the skin, soft tissues, upper airways, bones, and central nervous system. Diagnostic immunohistochemistry typically demonstrates S-100 protein positivity in histiocytes [4].

There is no standardized treatment for RDD. Management is individualized based on the extent and severity of the disease. Spontaneous remission is reported in 20-50% of the cases. Surgical excision can be curative for isolated lesions. Other therapeutic modalities, including corticosteroids, chemotherapy, radiotherapy, immunomodulatory agents, and Sirolimus, have been employed with varying success [5].

Herein, we present a pediatric case of RDD diagnosed and managed in a Pediatrics Department in the Oriental Region of Morocco.

## Case presentation

**Figure 1:**
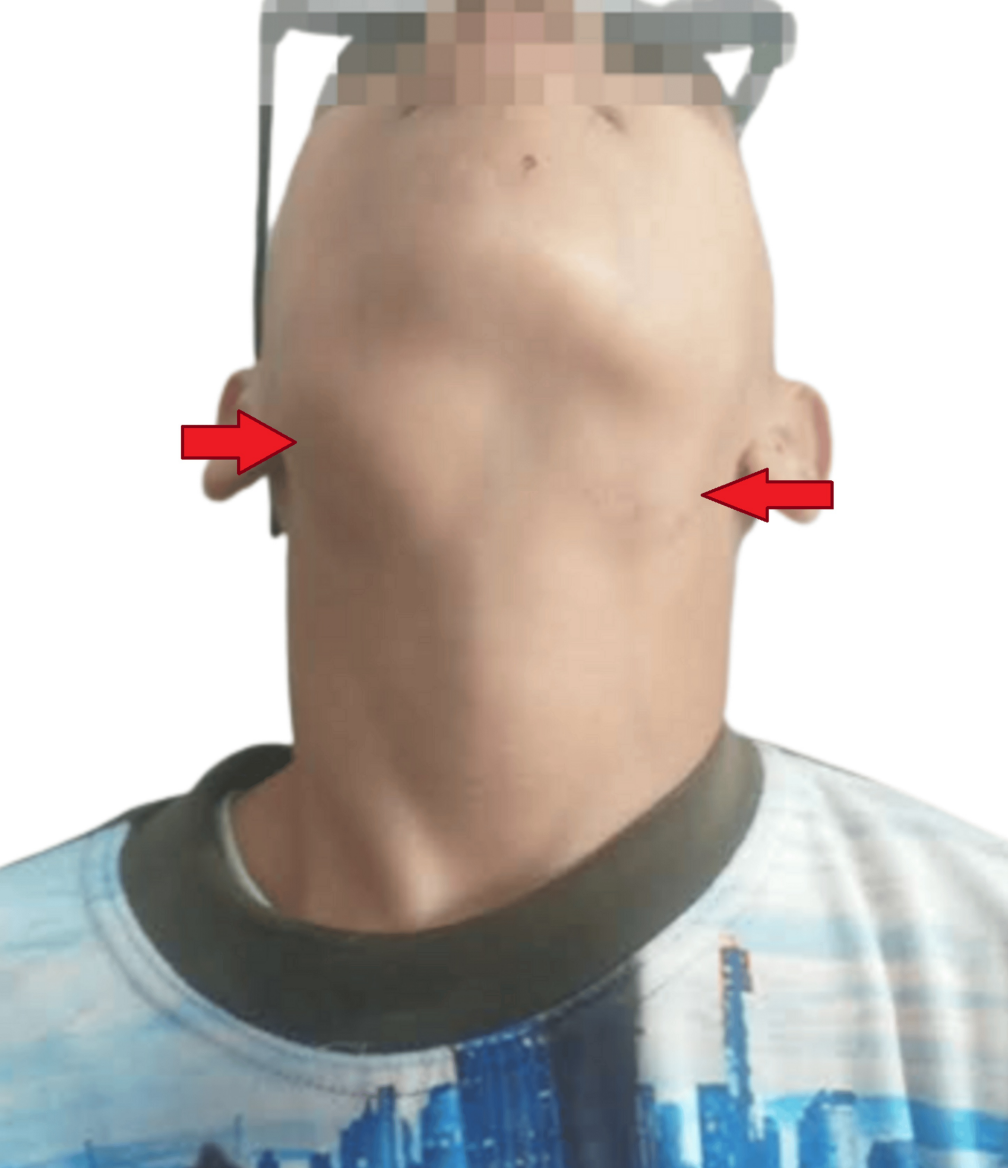
Enlarged bilateral cervical lymph nodes upon admission This highlights the initial clinical feature before the progression of the disease.

This report concerns a 12-year-old patient with a medical history that began seven months before admission. Initially, the patient presented with a painful swelling under the left mandible, which gradually increased in size without any signs of inflammation or other symptoms. Two months after the onset of symptoms, the patient consulted an otolaryngologist, who performed an excisional biopsy of the mass. Histopathological analysis of the biopsy indicated the absence of malignancy. However, one week following the procedure, the patient noted the appearance of a new mass in the right mandibular area (Figure 1). This mass also progressively increased, leading to another consultation with an otolaryngologist. A rhinoscopy and biopsy of a mass in the left nasal cavity were performed, and histopathological findings were consistent with Rosaï-Dorfman-Destombes disease. Consequently, the patient was referred to our department for further management. 

Upon clinical examination, the patient appeared in good general condition. Physical examination revealed multiple enlarged bilateral cervical and submandibular lymph nodes without any restriction in neck movement. Palpation of the neck swelling revealed firm, mobile, non-tender masses, the largest of which measured 8x10 cm. Systemic examination showed no hepatosplenomegaly, and the cardiovascular, respiratory, and nervous systems were unremarkable. Laboratory bioassays were within normal limits, and extended serological tests returned negative results for active infections. During hospitalization, computed tomography (CT) scans of the chest and abdominopelvic regions were performed, which revealed no additional nodal involvement.

**Figure 2:**
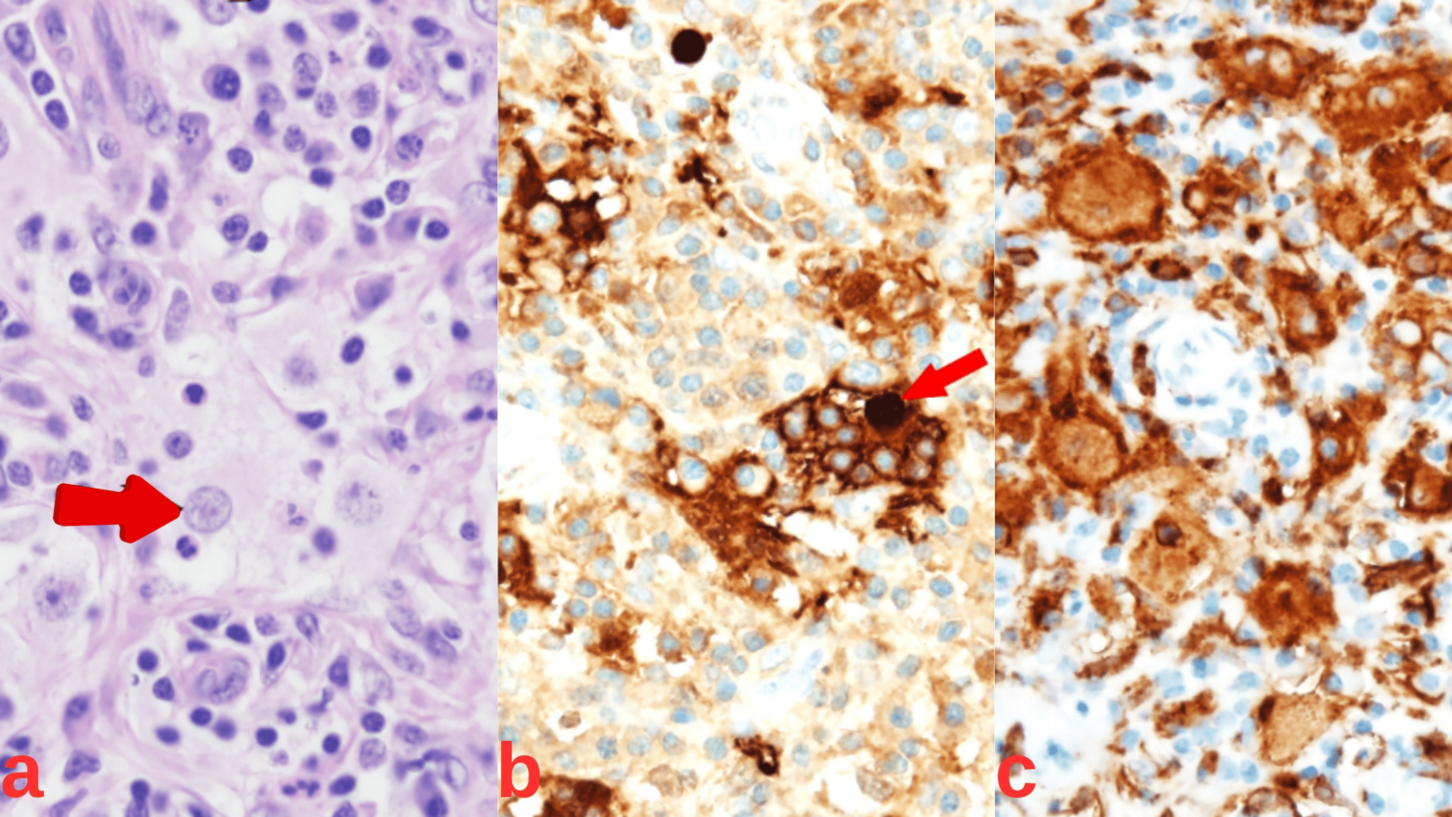
Histological sections of the excised lymph node demonstrating increased volume. (a) Histiocyte exhibiting emperipolesis with numerous engulfed lymphocytes; (b) Immunohistochemical staining highlighting strong S100 protein expression; (c) Immunohistochemical staining highlighting strong CD163 expression.

Histopathological examination of the biopsied tissue demonstrated the presence of emperipolesis and significant sinus infiltration by large histiocytic cells with pale cytoplasm. Immunohistochemical analysis revealed that these cells were positive for CD163 and S-100 protein, and negative for CD1a, which confirmed the diagnosis of Rosaï-Dorfman-Destombes disease (Figure 2).

Following the diagnosis, the patient was discharged after four days with a prescription for oral prednisone at a dosage of 1 mg/kg/day. The prednisone therapy was gradually tapered over 30 days. Initial clinical follow-up showed a significant reduction in cervical lymphadenopathy. However, due to subsequent local progression, the patient was treated with 6-mercaptopurine and methotrexate. Further clinical follow-up showed a marked reduction in cervical lymphadenopathy following this treatment regimen.

In summary, this case highlights the diagnostic and therapeutic challenges associated with Rosaï-Dorfman-Destombes disease, emphasizing the importance of a multidisciplinary approach for effective management. The patient's response to treatment underscores the need for continued monitoring and potential adjustment of therapeutic strategies to ensure optimal outcomes.

## Discussion

Rosaï-Dorfman-Destombes disease (RDD), also known as sinus histiocytosis with massive lymphadenopathy (SHML), is a rare and benign histiocytic disorder first clinically described by Rosaï and Dorfman in 1969. It is characterized by a proliferation of histiocytes, primarily within lymph nodes, although extranodal involvement is not uncommon. Despite its benign nature, RDD can present with diagnostic and therapeutic challenges, as demonstrated in this case [6].

The etiology of RDD remains poorly understood, although infectious and immune-mediated mechanisms have been proposed. The role of viruses, such as Epstein-Barr virus (EBV), human herpesvirus-6 (HHV-6), and parvovirus B19, has been investigated, but definitive evidence linking these agents to the disease is lacking. The hypothesis that viral infections may induce reactive histiocytic proliferation is intriguing but unproven. In this case, extensive serological investigations failed to identify an active infectious trigger, which is consistent with many reported cases [7].

The patient presented with the hallmark feature of RDD: painless cervical lymphadenopathy. However, the progression to submandibular lymph node involvement and the absence of systemic symptoms like fever or leukocytosis highlight the variability in clinical presentation. While fever and leukocytosis are common in RDD, their absence in this case emphasizes the importance of histopathological evaluation to differentiate RDD from other causes of lymphadenopathy, including lymphoma and infectious processes [8].

Histopathology remains the cornerstone of diagnosis. This patient’s lymph node biopsy revealed emperipolesis (intact lymphocytes within histiocytic cytoplasm), a pathognomonic finding in RDD, along with immunohistochemical positivity for S100 and CD68. These findings, coupled with negative markers for CD1a, ruled out alternative diagnoses such as Langerhans cell histiocytosis. The presence of pericapsular fibrosis and sinusoidal infiltration further supported the diagnosis [4,9]. 

Management of RDD is complex and varies based on the extent and severity of the disease. Spontaneous remission has been reported in up to 50% of cases, but active intervention is often required for symptomatic or progressive disease. This patient initially received corticosteroid therapy, which led to a significant reduction in lymphadenopathy. However, subsequent progression necessitated the addition of immunosuppressive agents, specifically low-dose methotrexate and 6-mercaptopurine. This combination has been documented as effective in refractory cases, and it yielded a favorable response in this patient [10].

Surgical intervention is typically limited to biopsy or debulking for symptomatic relief in cases involving critical structures. In this case, surgical debulking was not required due to the efficacy of medical management. Radiotherapy and chemotherapy are reserved for severe, refractory, or life-threatening cases, such as those involving the central nervous system or upper airway obstruction. While not needed in this case, these modalities remain important considerations in the therapeutic arsenal for RDD [5].

This case underscores the importance of a multidisciplinary approach to the diagnosis and management of RDD. The diagnostic process often involves clinicians, radiologists, and pathologists to differentiate RDD from other conditions that mimic its presentation. Moreover, long-term follow-up is essential given the potential for recurrence or progression, even in patients with initial clinical improvement. Pediatric cases, in particular, require careful monitoring to avoid treatment-related adverse effects and ensure optimal developmental outcomes. 

The variability in RDD presentation, pathogenesis, and response to treatment highlights the need for further research into its underlying mechanisms and management strategies. Advancements in genetic and molecular studies may provide insights into the etiology and potential targeted therapies for this rare disorder. This case contributes to the growing body of literature on RDD, emphasizing the need for individualized care and vigilance in both diagnosis and treatment [11].

## Conclusions

Rosaï-Dorfman-Destombes disease (RDD) is a rare and multifaceted condition that presents significant challenges in both diagnosis and treatment. Compared to other histiocytic disorders such as Langerhans cell histiocytosis (LCH) and Erdheim-Chester disease (ECD), the progress in understanding the biological and molecular mechanisms underlying RDD has been relatively limited. This highlights a critical need for further research into the genetic and molecular alterations associated with RDD and their potential implications for targeted therapies.

An effective management of RDD often necessitates a multidisciplinary approach. Thorough assessment, including detailed clinical history, comprehensive physical examination, advanced imaging techniques, and relevant laboratory investigations, is crucial to delineate the extent of disease involvement and identify potential comorbidities. While observation may suffice for some cases, others require tailored interventions involving immunomodulatory agents or antineoplastic therapies, emphasizing the need for individualized treatment strategies.
